# Chill-inducing music enhances altruism in humans

**DOI:** 10.3389/fpsyg.2014.01215

**Published:** 2014-10-28

**Authors:** Hajime Fukui, Kumiko Toyoshima

**Affiliations:** Faculty of Education, Nara University of EducationNara, Japan

**Keywords:** music, emotion, altruism, empathy, prosocial behavior, dictator game

## Abstract

Music is a universal feature of human cultures, and it has both fascinated and troubled many researchers. In this paper we show through the dictator game (DG) that an individual’s listening to preferred “chill-inducing” music may promote altruistic behavior that extends beyond the bounds of kin selection or reciprocal altruism. Participants were 22 undergraduate and postgraduate students who were divided into two groups, the in-group and the out-group, and they acted as dictators. The dictators listened to their own preferred “chill-inducing” music, to music they disliked, or to silence, and then played the DG. In this hypothetical experiment, the dictators were given real money (which they did not keep) and were asked to distribute it to the recipients, who were presented as stylized images of men and women displayed on a computer screen. The dictators played the DG both before and after listening to the music. Both male and female dictators gave more money after listening to their preferred music and less after listening to the music they disliked, whereas silence had no effect on the allocated amounts. The group to which the recipient belonged did not influence these trends. The results suggest that listening to preferred “chill-inducing” music promotes altruistic behavior.

## INTRODUCTION

On March 11, 2011, Japan experienced an unprecedented disaster in which many people lost their families and homes in an instant. However, in the midst of this terrible situation, we witnessed behavior again and again that seemed unusual. For survivors, who were in the depths of despair, it appeared that music had the power to inspire courage and cooperation in them. This has also been the case throughout the period since the disaster. Countless concerts have been held not only in the affected areas but throughout Japan and worldwide to mourn for those who lost their lives and express hope for recovery (e.g., The Japan Foundation, 2012). There are times when people sacrifice their own interests to help others, but such prosocial behavior is typically expressed through financial donations or voluntary work (Miller et al., 1991). Music does not usually appear to be associated with “prosocial behavior.”

As is widely known, music has the ability to strongly affect a person’s emotions and sometimes even control them (Juslin and Sloboda, 2010), though science has not yet provided an explanation for this phenomenon. Darwin (1871) noted the mysterious quality that lies in man’s capacity to create music (Fitch, 2006). Indeed, what is the true value of music, which fascinates many scientists, if it does not have an adaptive function or survival value? The ability of music to absorb and captivate people remains an enigma.

The emotions induced by music have attracted the attention of numerous researchers (Budd, 1985; Juslin and Sloboda, 2010). In addition, it has been shown that music can arouse various complex emotions that are both positive and negative (Trost et al., 2012) as well as either pleasant or unpleasant (Koelsch et al., 2006). However, studies regarding emotion and music have primarily focused on listening and there are limited studies that have compared emotions evoked by performing and listening to music. According to the study by Nakahara et al. (2011), by modulating emotion-related autonomic nerve activity, musical performance can be more effective than musical perception (listening) in musicians. However, it remains unclear whether there is difference between emotion evoked by performing or listening. Recent studies have revealed that the emotions evoked by listening to music can modulate activity in all the limbic and paralimbic brain structures, and that those emotions tend to be stronger than everyday feelings or moods; therefore, they are registered as a strong experience (Koelsch et al., 2010) and are associated with particular brain activity (Koelsch et al., 2006).

It has been proposed that human altruism is engendered by empathy (Silka and Housea, 2011). If, as the above research suggests, emotional changes elicited by music activate empathy circuits in humans, music would also be expected to enhance altruistic behavior.

Several studies have examined music and altruism, or prosocial behavior (Kirschner and Tomasello, 2010; Kokal et al., 2011). Joint drumming in a social condition (drumming together with a partner) facilitates synchronization in preschool children and elicits a specific human motivation to synchronize movements during joint rhythmic activity (Kirschner and Tomasello, 2009). In addition, music making, including joint singing, encourages participants to maintain a collective intention and shared goal of vocalizing and moving together in time, thereby effectively satisfying the intrinsic human desire to share emotions, experiences, and activities with others (Kirschner and Tomasello, 2010). Several studies have indicated that music listening can mediate several behaviors related tangentially to altruism, such as maintaining low levels of classroom noise (Wilson and Hopkins, 1973), aggression (Konečni et al., 1976), and spending money in the school cafeteria (North and Hargreaves, 1998).

Listening to music can be a highly rewarding experience for humans. Although there has been little research in the field of music psychology, psychology, and neuroscience, studies have shown that a positive mood increases prosocial behaviors. Empirical evidence has revealed the effects of moods on behavior and many studies have shown that moods can affect a person’s behavior (Wright and Bower, 1992; Gendolla, 2000) or decision (Schwarz, 2000; Lerner et al., 2004; McEwen, 2012). There have also been reports that both positive and negative moods promote altruism and prosocial behaviors (Batson, 1991; Dovidio et al., 2006). It has been found, for example, that audiotapes can induce positive moods, thereby increasing altruism and thus giving rise to prosocial behavior (economic games; Capra, 2004). Prosocial songs were associated with a significant increase in tipping behavior (Gueguen, 2001). Fried and Berkowitz (1979) found that soothing (classical) music promoted greater levels of altruism (offering help to others) than did either aversive music (such as modern jazz) or no music. Anshel and Kipper (1988) reported that group singing (popular songs) promoted trust and cooperation among participants in an experiment. North et al. (2004) found that uplifting music (background music) led to participants’ offering more help on an expensive, leaflet-distributing task, than did annoying music.

There are few studies, however, that have directly investigated the relationship between the emotions evoked by music (strongly positive or negative emotions) and prosocial behavior or altruism. Similarly, no study related to music has focused specifically on the dictator game (DG). The DG shows the altruistic tendency most clearly, and it is the most well-known paradigm, one used frequently by economists, to test the existence of altruism (e.g., Bolton et al., 1998; Camerer and Fehr, 2006; Knafo et al., 2008; Henrich et al., 2010). We used the DG (with music as the background stimulus condition) in order to explore the relationship between the emotions evoked by listening to music and altruistic tendencies.

## MATERIALS AND METHODS

### PARTICIPANTS

Participants in this double-blind experiment were 11 healthy men (aged 19–23 years, mean age 21 years) and 11 healthy women (aged 19–23 years, mean age 21 years), mainly recruited from among undergraduate and postgraduate students. Because it has been demonstrated that the menstrual cycle of women influences their behavior in economic games (Pin and Fletcher, 2011), the experiment was conducted in the follicular phase (within 6 days after the end of menstruation) for the female participants to standardize this variable. All gave written informed consent to participate in this study based on the Declaration of Helsinki (2000) and the University Research Ethics Standards.

Since this study examines the overall impact of listening to music on altruism, we eliminated the professional musicians. The remaining subjects were considered to be those from the general population who had an average of 9.9 years of experience in music (instrumental or vocal) either through music education classes or individual study. We conducted a preliminary survey to investigate whether potential participants had ever experienced “chills,” a powerful music-induced emotional experience, which is used by many researchers as a subjective indicator of the experience of feeling music (e.g., Gabrielsson, 2001; Hodges, 2010; Nusbaum and Silvia, 2010). Only those who responded affirmatively were selected as participants.

### BRIEFING SESSION

In the briefing session, participants completed a number of tests and a questionnaire that addressed their musical tastes and emotional experiences through music. Questionnaires included the NEO Five-Factor Inventory (NEO-FFI), the Cambridge Behavior Scale (Empathy Quotient; EQ), and the State-Trait Anxiety Inventory (STAI-state and STAI-trait).

The NEO-FFI is a shortened version of the NEO Personality Inventory-Revised (NEO-PI-R), which measures five domains of personality: openness to experience, conscientiousness, extroversion, agreeableness, and neuroticism. The NEO-FFI measures these domains using a 60-item questionnaire and a 5-point response scale from “strongly agree” to “strongly disagree” (Costa and McCrae, 1992). The Cambridge Behavior Scale measures scores on empathy (EQ). This test is comprised of three subscales: cognitive empathy, emotional reactivity, and social skills. It uses a 60-item self-reporting, 4-point scale ranging from “strongly agree” to “strongly disagree” (Baron-Cohen and Wheelwright, 2004). The STAI consists of Form I, which measures state anxiety, and Form II, which measures trait anxiety. Each form contains 20 items, for a total of 40 items (Spielberger et al., 1970). Participants responded on a 4-point scale from “very much” to “not at all.” The STAI-I was administered before and after the stimulus presentation in this experiment.

We analyzed the correlation between the preliminary survey scores for the NEO-FFI, the EQ, and the STAI questionnaires and the changes in allocated amounts in the DG before and after stimuli, but found no significant influences.

### DICTATOR GAME

Participants were informed of the basic rules and the concept of the game prior to the experiment. They were divided into two groups of 11 members each, according to the lots they drew, and the groups were named the in-group (IG) and the out-group (OG), the dictators and the recipients, respectively. They were also told that every participant in each group would take part in the DG as dictator.

The participants then assembled with the members of their group, facing a computer screen that displayed a mark identifying their group (a vertical or horizontal line). This was done to make the participants aware that there were two different groups and to heighten their sense of belonging to their own group. Also, in order to encourage the participants to self-identify and feel a strong social identity with their groups, they wore T-shirts with logos of their group on the front, which clearly indicated who belonged to the IG and who belonged to the OG (Gino et al., 2009).

Each participant played the dictator’s role in the DG. There were four recipients in total: one male and one female who belonged to the IG, and one male and one female who belonged to the group OG.

**FIGURE 1:**
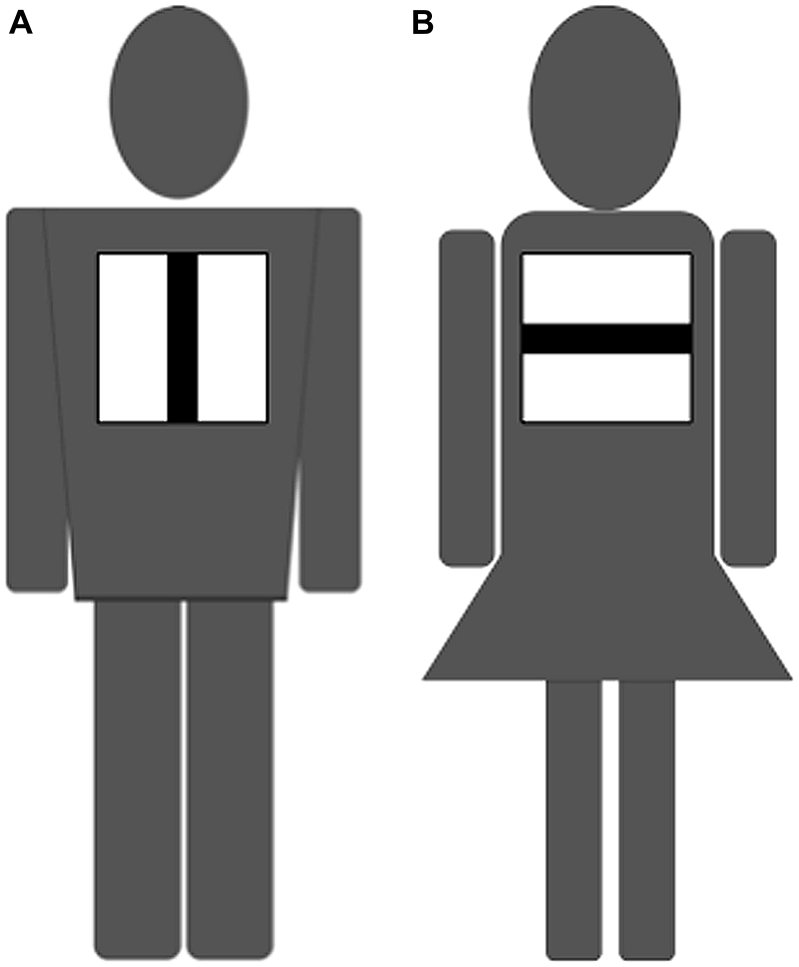
**Stylized recipient in Dictator Game (DG).** These images are recipients in the DG experiment, displayed on a 19-inch screen in front of the dictator. Four types of recipients were created through a combination of the recipient’s sex (man or woman) and group membership (vertical group or horizontal group). **(A)** One of the four patterns: a man belonging to the vertical group. **(B)** A woman from the horizontal group.

In the DG, information about the recipients (e.g., attractiveness) can affect the decision of the dictator (Aguiar et al., 2008; Rosenblat, 2008); therefore, we eliminated these factors as much possible by ensuring that the dictators did not see the recipients. The recipients in the experiment were stylized images of male and female humans shown as black-and-white figures, whose only identifying features were sex and group, thus eliminating the factor of attractiveness. These images were displayed on a 19-inch computer screen placed in front of the dictators (**Figure 1**).

This experiment was hypothetical, as the dictators were given real money, but were not permitted to keep the money. Previous studies have shown that reward type (real or hypothetical) made no significant difference in cooperation. The dictator will allocate money even if the experimenters do not use pecuniary incentives, and that the effect of the manipulation is insignificant (e.g., Aguiar et al., 2008; Ben-Ner et al., 2008; Engel, 2011; Locey et al., 2011; Radke and de Bruijn, 2012).

### MUSIC

Musical emotions are induced through a complex interaction between the music, the listener, and the environment, and are influenced by factors such as personality and culture (Sloboda and Juslin, 2010). A variety of methods have been used to investigate musical emotions, but one highly reliable approach centers around the concept of “chills,” which many researchers use as a subjective indicator of musical feeling (e.g., Hodges, 2010; Nusbaum and Silvia, 2010; Balteş et al., 2011). “Musical chills” are a phenomenon involving strong affective changes such as tears, shivers down the spine, and goose bumps (Gabrielsson and Lindström, 2003). However, not everyone experiences musical chills (Salimpoor et al., 2009). For this reason, we recruited individuals who had experienced “chills” as an intensely pleasurable response to music (Salimpoor et al., 2009), as noted earlier.

For this experiment, we asked the participants to choose two types of music in advance: (1) music they preferred (chill-inducing) and (2) music they did not like. We used silence as the third condition. The participants took part in all sessions (3 stimuli × 4 games). In this experiment, the same musical pieces (preferred and disliked) were repeated four times (same sex: IG and OG, opposite sex: IG and OG), and the orders were randomized. Until the start of the experiment, the participants were not informed which stimulus would be presented or with whom they would play the DG.

The three categories of stimulus were each presented for 5 min, and the participants who had selected music lasting more than 5 min were asked to choose a 5-min passage that contained the most chill-inducing or most disliked part within the piece. About half of the music that the participants chose as preferred music (chill-inducing) was Japanese pop, and the remainder included classical piano and orchestral music. Disliked music included contemporary music, traditional Japanese music, *Enka* (sentimental ballads), and computer game music. There were a roughly equal number of vocal and instrumental pieces.

The participants listened to the music on headphones. Stimuli were presented as MP3 files (sample-rate 44.100 kHz) that were played on a computer. For the silent control condition, participants wore headphones in the same way as when listening to music.

### EXPERIMENTAL PROCEDURE (FIGURE 2)

On the day of the experiment, participants took part in the DG sessions. Participants were first instructed to put on the T-shirts printed with a logo that identified their group to heighten their sense of group belonging. They then completed the STAI-state and played the DG before and after the stimulus presentation (preferred music, disliked music, or silence). At the end of each session, participants were asked to rate their feeling of “chill” toward the stimulus on a 7-point scale from “completely unmoved” to “highly moved.” Simultaneously, participants expressed what and how they felt during the experiment through freewriting. Each session lasted about 20 min. In order to avoid the influence of an experimenter’s sex, the experimenter who gave the instructions was the same sex as the participant.

**FIGURE 2:**
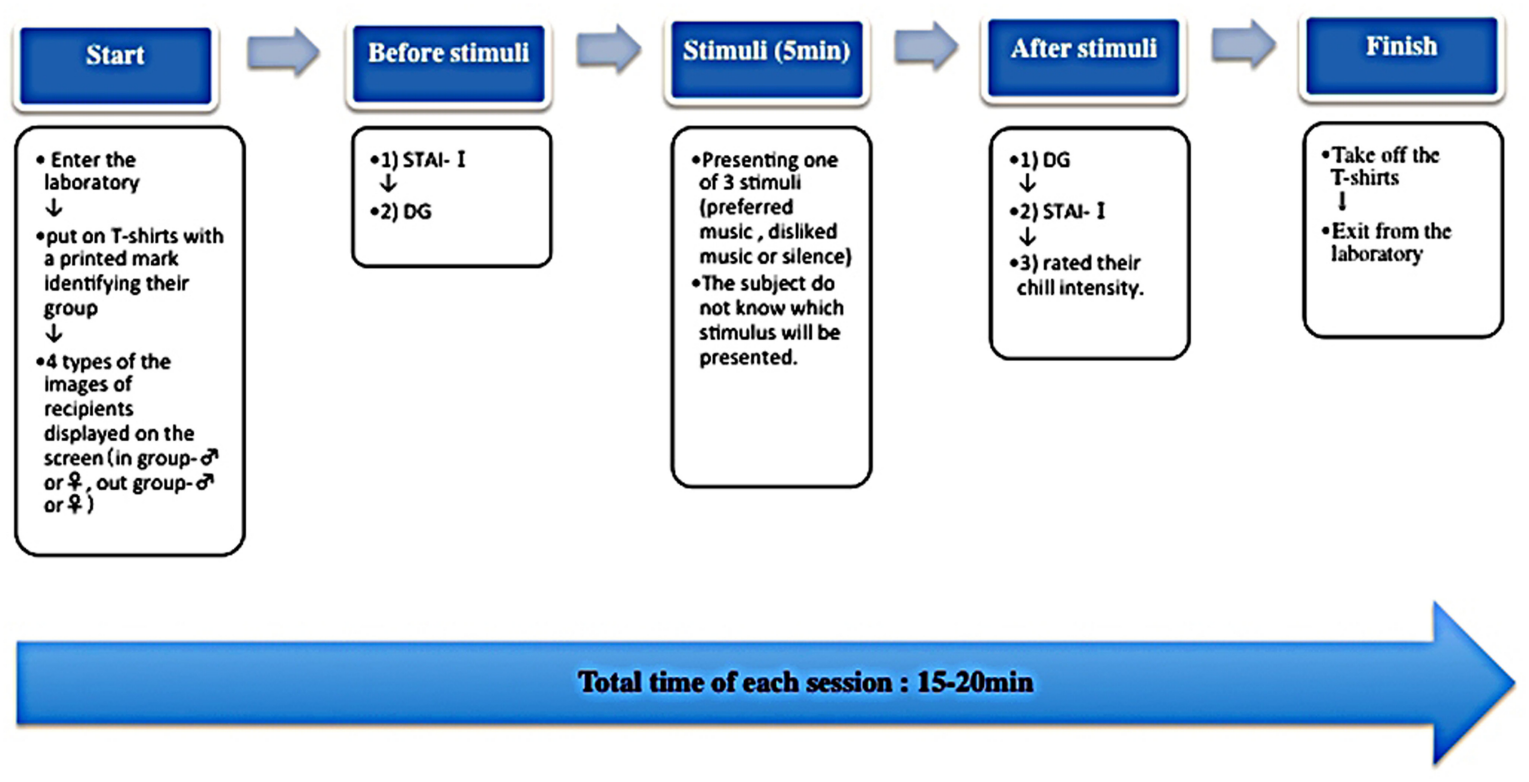
**Sequence of events in the experiment.** The above diagram shows the flow of a single session. The time required for one session was about 20 min. All participants participated in 12 sessions (3 stimuli: preferred music, disliked music, and silence × 4 DG games). Sessions were separated by rest periods of at least 30 min in order to counteract the effects of the previous session. The participants participated in 3–4 sessions per day, and the experiment was carried out over 3–4 separate days.

At the start of each game, the dictator was given cash (10,000 Japanese yen) and instructed to allocate this fund between him-/herself and the recipient in units of 1,000 yen. Before making the allocation, the dictator was discreetly told that as the dictator, it was up to him/her what to do with the money. The allocation was entirely at the dictator’s discretion. The recipient had no right to receive or to refuse the allocation, and therefore the dictator was entirely free to give money to the recipient or not. While the recipient was displayed on the computer screen, the dictator checked and verbally reported to the experimenter whether the recipient was a man or a woman, and whether the recipient belonged to the same group as the dictator or not. Dictators were then instructed to allocate the money. Before the participant’s first actual game, we administered two training games and checked whether the participant understood the rules.

To avoid interference, the experimenter withdrew to another room before the allocation so that the dictator could decide how much to give at his/her own discretion. In addition, the dictator directly put the divided cash into a cash box and locked it with a dial lock so that the experimenter could not know the allocated amount.

Each participant played the game in his/her assigned role as the dictator with each of the four recipients (one man and one woman of the IG; one man and one woman of the OG) under each of the three stimulus conditions (two types of music, or silence), for a total of 12 sessions. In the control condition, the procedure was exactly the same as the music condition except that the dictators did not listen to any music (i.e., silence was used).

Sessions were separated by rest periods of at least 30 min to avoid carryover effects (fatigue). Also, to help the participants avoid fatigue, the experiment was carried out in 3–4 sessions per day over 3–4 separate days. In order to avoid the order effect, the presentation of stimuli (3 stimuli × 4 games) was randomized.

As noted previously, in the DG, information about the recipients (e.g., attractiveness) can affect the decision-making of a dictator (Aguiar et al., 2008; Rosenblat, 2008). Therefore, to eliminate these effects as much as much possible with regard to who played the music, after the experiment, the dictators were asked a multiple-choice question: “who was playing the music?” The possible responses were: (1) the dictator, (2) the recipient, or (3) the music was played in the setting where the DG was carried out. None of the dictators thought that they themselves or the recipients had performed the music, and believed that it was played in the environment where the DG was carried out. Therefore, the researchers were assured that any bias was eliminated.

## RESULTS

**FIGURE 3:**
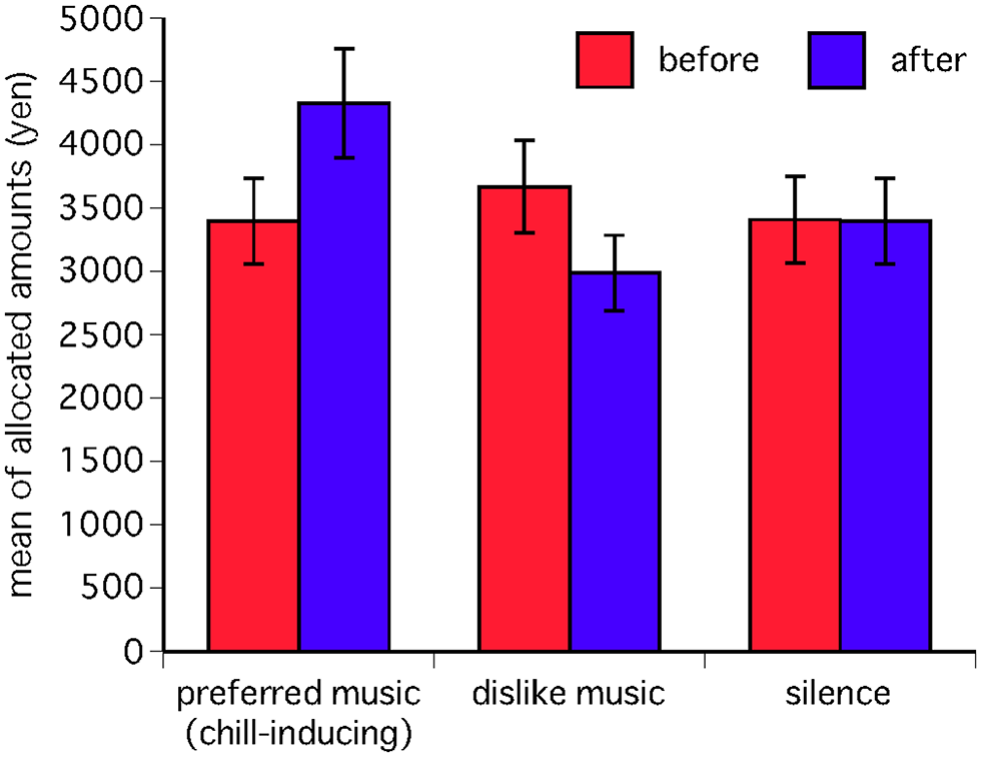
**Changes in allocation of DG money before and after stimulus.** This graph shows changes in the amounts allocated to recipients before and after presentation of three types of stimuli (preferred music, disliked music, and silence) in a DG experiment played by 11 male and 11 female participants (Error bars show SD). The amount allocated to recipients significantly increased after participants listened to their preferred music, but decreased after they listened to their disliked music [*F*(2,240) = 69.019, *p* = 0.000].

The DG (allocation) was analyzed through a four-way repeated-measures analysis of variance (ANOVA) with the following factors: “music” (chill-inducing, dislike, silence as control), “recipient’s group” (IG, OG), “recipient’s sex” (male, female), and “subject’s sex” (male, female). As a measure of effect size ETA^2^ is reported. Regarding a significant multivariate effect, *post hoc* paired *t*-tests were computed using the Bonferroni correction. The results showed that allocation behavior before each of the three stimuli was the same as the behavior that is consistently seen in past studies of the DG (Engel, 2011). However, the present experiment showed that the allocation was significantly different after stimuli presentation, depending on whether the stimulus was silence, preferred (chill-inducing) music, or disliked music. The average DG-allocations are presented in **Table 1**. The ANOVA (DGs × 3 stimuli) revealed a significant interaction [*F*(2,240) = 69.019, *p* = 0.001, ETA^2^ = 0.6] that both male and female dictators gave more money after listening to their preferred music and less after listening to their disliked music (Bonferroni *p* = 0.009), whereas silence had no effect on the allocated amounts (**Figure 3**). The group to which the recipient belonged did not influence these tendencies. However, the ANOVA (DGs × group) revealed that the actual amount that was allocated differed depending on whether the recipient belonged to the IG or the OG [*F*(1,240) = 57.679, *p* = 0.001. ETA^2^ = 0.3; Bonferroni *p* = 0.001]. Male dictators also gave higher amounts than female dictators (DGs × sex) [*F*(1,240) = 16.141, *p* = 0.001, ETA^2^ = 0.1; Bonferroni *p* = 0.001]. The STAI-state (STAI points × 3 stimuli) revealed a significant interaction [*F*(2,240) = 28.196, *p* = 0.001, ETA^2^ = 0.3] that preferred music decreased anxiety, while disliked music increased anxiety (Bonferroni *p* = 0.001). We analyzed the changes in allocated amounts in the DG to see if they were affected by the presence or absence of words in the music, but ANOVA revealed no significant differences. Participants chose both vocal and instrumental music, but the ANOVA revealed no correlation between the results for either the type of music or the level of musical experience.

**Table 1:** Mean allocation of each stimuli.

Subjects	Stimuli	Recipients sex	Recipients group IG, In-group; OG, Out-group	Before Mean (Yen)	After Mean (Yen)	SD	SD
Male (*n* = 11)	Chill-inducing music	Male	IG OG	4181.82 2909.09	981.65 1445.998	4909.09 3636.36	831.209 1361.817
		Female	IG OG	4363.64 3454.55	1120.065 1368.476	5000 3818.18	1341.641 1328.02
	Disliked music	Male	IG OG	4727.27 3363.64	786.245 1286.291	4000 3000	1341.641 1264.911
		Female	IG OG	4454.55 3454.55	1128.152 1439.697	4181.82 3272.73	1250.454 1190.874
	Silence	Male	IG OG	3909.09 3090.91	1445.998 1513.575	3727.27 3272.73	1420.627 1420.627
		Female	IG OG	4727.27 2909.09	904.534 1513.575	4636.36 2818.18	809.04 1662.419
Female (*n* =11)	Chill-inducing music	Male	IG OG	3545.45 2363.64	1128.152 1120.065	4545.45 3818.18	1213.56 1778.661
		Female	IG OG	3909.09 2454.55	1136.182 1368.476	4454.55 4454.55	1213.56 1752.92
	Disliked music	Male	IG OG	3636.36 2636.36	1206.045 1286.291	2909.09 1818.18	1445.998 1401.298
		Female	IG OG	4000 3090.91	774.597 1136.182	3181.82 1545.45	1250.454 1293.34
	Silence	Male	IG OG	3545.45 2272.73	1507.557 1272.078	3454.55 2454.55	1439.697 1035.725
		Female	IG OG	4000 2818.18	1000 1250.454	4090.91 2727.27	700.649 1103.713

In a post-game session, a survey was taken, and all participants responded that they had experienced chills (highly moved: rating of 6 or 7 on a 7-point rating scale) when listening to their preferred music and were unaffected (completely unmoved: rating of 1 or 2 on a 7-point rating scale) when listening to their disliked music. No correlations were found with the NEO-EFI, EQ. There were no such comments found in what the participants wrote about their feelings during the experiment, which could have influenced the results.

## DISCUSSION

Decision-making in humans is strongly influenced not only by logic but also emotion (Coricelli et al., 2007). In the experiment, after listening to their preferred (chill-inducing) music, the dictators increased the amount of money allocated to the recipient regardless of whether the recipient was in the IG or OG, whereas they decreased the amount after listening to disliked music, no matter which group the recipient belonged to. The actual amount allocated differed between IG and OG recipients; dictators distributed more to the IG than to the OG. As noted, this experiment was hypothetical, though the results are consistent with a previous study (Forgas and Fiedler, 1996). However, after listening to music, the dictators showed no difference or preference, as seen in the allocation that they made. This shows that music can activate both altruistic behavior and selfish behavior. It is vital to note that music affects an individual’s behavior greatly; preferred music promotes altruistic behavior, whereas disliked music is associated with selfish behavior, and what differentiates these behaviors is the emotional response dictated by the listener’s musical preferences.

Emotions induced by music are thought to be associated with the action of mirror neurons and the limbic system (Zatorre and McGill, 2005). Thus, the internal experience of music may be mediated by the human mirror neuron system (Overy and Molnar-Szakacs, 2009). Studies involving musicians and dancers have also reported that the internal experience of music modulates the activity of the human mirror neuron system (D’Ausilio et al., 2006). Mirror neurons have been linked to the higher brain function of empathy, and it has been suggested that the emotional response to music is also an empathic process resulting from listening to a performance by other humans (Chapin et al., 2010). Many reports have indicated that the mirror neuron system is related to empathy (Overy and Molnar-Szakacs, 2009; Decety, 2011), which is also a central mechanism of music-induced emotions (Juslin et al., 2010). However, even though the presence of the inferior frontal cortex and superior parietal lobe is assumed, the existence of mirror neurons in the human brain has not yet been confirmed.

There are many neurophysiological studies that have shown how visual stimuli evoke emotions. Moreover, there are reports that strong emotion enhancement affects the simultaneous presentation of congruent emotional pictures and music (Baumgartner et al., 2006, 2007). In addition, visual bias (attractiveness) induces IG favoritism in behaviors (Ratner et al., 2014). As stated earlier, in the DG, information about the recipients (e.g., attractiveness) can affect the decision of the dictator (Aguiar et al., 2008; Rosenblat, 2008). In this regard, we applied stylized (black-and-white) images of males and females whose only identifying features were sex and group, to eliminate the factor of attractiveness. Thus, it is believed that the results were caused by music with strong emotions (chill-inducing music) that was presented concurrently with such visual information.

Research on the affective influences of altruism and helping behavior has remained largely inconclusive. Studies suggest that both positive and negative effects can promote altruism and prosocial behaviors, and that the results depend upon the circumstances (Dovidio et al., 2006). In the present experiment, music elicited emotional reactions (decreasing or increasing anxiety: STAI-state), which can be interpreted as positive and negative moods (Rossi and Pourtois, 2012). A positive mood induced by preferred music promoted altruistic behavior, whereas a negative mood induced by disliked music increased selfish behavior. This result does not match the previous finding that a negative mood can also increase altruistic behaviors (Capra, 2004; Tan and Forgas, 2010). Musical emotions are difficult to define scientifically, but at the very least, we can say that preferred music elicits chills, while disliked music does not. That is to say, the emotion evoked by musical stimuli (chill) is distinct from everyday emotion (mild mood; Patel, 2008), and it causes strong emotional responses. It may be that the dictators behaved altruistically toward others because they experienced positive musical emotions, which activated the brain’s reward system, which is centered in the limbic system, and simultaneously activated the empathy circuits. Key brain regions for processing musical emotions are the striatum, the amygdala, the orbitofrontal cortex, and the anterior cingulate cortex (Zatorre and McGill, 2005).

In contrast, self-interested behavior may have occurred as a result of the dictators’ listening to disliked music. The aversive stimulus may have induced negative musical emotions that activated other reward circuits (the ventromedial orbitofrontal cortex and the amygdala, the ventral striatum, and the ventromedial prefrontal cortex; Tricomi et al., 2010). The amygdala appears to be necessary for the emotional processing of music (Gosselin et al., 2007; Rand et al., 2012).

Strong emotions such as chill responses to music are linked to an activation of the sympathetic nervous system and the brain reward circuits. The chill response occurs in the auditory domain in the context of highly pleasurable events (music) that lead to the activation of the dopaminergic reward system in the brain (D’Ausilio et al., 2006). Salimpoor et al. (2009) found that there is an endogenous dopamine release in the striatum at peak emotional arousal during music listening (“chills”). Conversely, sad music, in contrast to neutral music, activated the hippocampus and the amygdala, consistent with the role of these structures in negative emotion perception (Gosselin et al., 2007; Lerner et al., 2009), and the cerebellum (Brattico et al., 2011). Increasing chill intensity decreases regional cerebral blood flow in the amygdala (Blood and Zatorre, 2001; Matthews et al., 2009).

Again, the amygdala is necessary for developing and expressing normal interpersonal trust (Koscik and Tranel, 2011). A study has shown that when the amygdala is damaged in an individual, that person invests more money with other people who are unfamiliar than do healthy controls (van Honk et al., 2013). Therefore, in this experiment, suppression of amygdala activity by chill-induced music might lead to a dictator’s increasing the distribution of money.

In the experiment, male dictators gave higher amounts than did female dictators regardless of the type of music, relationship to the recipients, or sex of the recipients. Research on sex (gender) differences in generosity or prosocial behavior has been contradictory. It is widely believed that females are more sensitive than males to “costs” (Riyanto and Zhang, 2010). Although both sexes prefer direct cooperative behavior toward more attractive members of the opposite sex (Farrelly et al., 2007), this result was easily predictable. The recipients of the experiment were therefore stylized images of male and female humans, whose only identifying features were sex and group, thus eliminating the factors of attractiveness. Our results, therefore, can be considered as consistent, and they were not affected by the appearance of the recipients. Males’ showing more generosity corresponds to the results of previous studies.

Musical ability is known to have a genetic component (Wallin et al., 2000), but the environment in the form of cultural variation also has a great influence. In essence, musical preferences are probably influenced by genetics and experience, but the degree of cultural difference, and diversity in music is probably much greater today than for early humans, who lived in small groups based on kinship. For them, the music of one’s group would probably have been the music an individual preferred and found emotionally moving. If a total stranger performed this preferred music, it would suggest a kinship or some other connection to one’s own group. It would therefore be rational to treat the stranger altruistically. Similarly, disliked music would suggest a weak connection and would justify selfish behavior. In our experiment, the recipients were identified by a logo indicating which group they belonged to. After listening to their preferred music, dictators treated the recipients wearing IG logos altruistically, but after the dictators heard disliked music, their behavior was selfish. Despite the dictators’ being in the same group as the recipients, the emotions caused by music led to changes in their allocation. The dictators also behaved altruistically toward the recipients in the OG after listening to preferred music. This behavior was somewhat illogical. Considering that in the pre-stimulus DG, dictators allocated money in the usually observed way (Forsythe et al., 1994; Bolton et al., 1998), this altruistic action toward the other group is remarkable.

All dictators thought that the music was being played in the setting in which they were facing their recipient. In this situation, the dictators would not have made their decisions on the basis of reciprocal altruism, because of the low probability of getting a reward from an OG recipient. Thus, the decisions that the dictators made after listening to their preferred music (which can be construed as listening to music played by members of the IG) might explain a unilateral altruistic behavior toward the OG. Tomasello et al. (2005) suggested that humans have a species-unique motivation to share emotions, experiences, and activities with other persons. Merker (2000) suggested that synchronous chorusing by hominid males served as an indicator of coalition strength, helping to defend territory and at the same time attract migrating females. Hagen and Bryant (2003) proposed that group music making (and dancing) evolved as between-group displays signaling internal stability and the group’s ability to act collectively, thereby establishing meaningful relationships—whether cooperative or hostile—between groups. Kirschner and Tomasello (2010) asserted that music (making) has a positive effect on prosociality and that its effects can be interpreted as evidence against the hypothesis that today’s music (and dance) are completely non-adaptive byproducts of the human mind. Our results showed that preferred music can promote cooperative behavior between groups.

Altruism is a recognized feature of human society, and it goes far beyond that which has been observed in the animal world (Fehr and Fischbacher, 2003), and extends beyond reciprocal altruism and reputation-based cooperation, taking the form of strong reciprocity (Gintis, 2000; Fehr et al., 2002). Musical emotion seems to play a role in regulating human decision-making and social behavior, but perhaps music itself evolved to promote group cohesion and social bonding. In any case, music is an emotional system arising through natural selection, yet it has undoubtedly become deeply connected to human social behavior. This study supports the hypothesis that music exists to promote socialization and maintain social cohesion, and is not merely an evolutionary byproduct or noise. This research also moves us a step closer toward elucidating not only the evolution and the biological function of music but also the evolution of human altruism.

## Conflict of Interest Statement

The authors declare that the research was conducted in the absence of any commercial or financial relationships that could be construed as a potential conflict of interest.
